# Poly (lactic-co-glycolic acid)-encapsulated iodine-131 nanoparticles fabricated with rhTSH induce apoptosis and immobilization of thyroid cancer cells

**DOI:** 10.3389/fonc.2023.1030105

**Published:** 2023-01-27

**Authors:** Yongzeng Fan, Yalan Xiong, Xinhong Wang, Jiahao Chen, Danzhou Fang, Jiahui Huang, Gengbiao Yuan

**Affiliations:** ^1^ Department of Nuclear Medicine, The Second Affiliated Hospital of Chongqing Medical University, Chongqing, China; ^2^ Department of Emergency, The Second Affiliated Hospital of Chongqing Medical University, Chongqing, China

**Keywords:** thyroid cancer, rhTSH, PLGA, iodine-131, apoptosis

## Abstract

**Background:**

Aggressive thyroid carcinoma (ATC) usually loses radioiodine avidity to iodine-131 (^131^I) due to the downregulation of sodium/iodide symporter (NIS). The expression of thyroid stimulating hormone receptor (TSHR) is more persistent than NIS and the administration of recombinant human thyroid stimulating hormone (rhTSH) promotes de novo NIS synthesis. Hence, exploring methods integrating ^131^I with rhTSH might be a feasible therapeutic strategy for selective delivery of ^131^I into thyroid cancer to fortify the effect of radioiodine ablation.

**Methods:**

The ^131^I, poly (lactic-co-glycolic acid) (PLGA) and rhTSH were used to synthesize of the ^131^I-PLGA-rhTSH nanoparticles. The characteristics of the ^131^I-PLGA-rhTSH nanoparticles was determined using a light microscopy, scanning electron microscopy (SEM), autoradiography and immunofluorescence (IF) staining. The diameter of the ^131^I-PLGA-rhTSH nanoparticles was measured with a Mastersizer 3000, and the encapsulation efficiency (EF) of ^131^I in ^131^I-PLGA-rhTSH nanoparticles and the radioactivity of a single nanoparticle were determined. Then, the mouse tumor xenograft model was established, and the biodistribution and effect of ^131^I-PLGA-rhTSH nanoparticles on apoptosis of thyroid cance cells were investigated in vivo. Thereafter, the role of ^131^I-PLGA-rhTSH nanoparticles in cell viability using cell counting kit-8 and lactate dehydrogenase (LDH) release assays. Subsequently, the underlying mechanism of ^131^I-PLGA-rhTSH nanoparticles in reducing cell viability was assessed using immunostaining, boyden invasion assays and phalloidin staining.

**Results:**

Our results showed that the method of developing nanoparticles-encapsulated ^131^I using poly (lactic-co-glycolic acid) (PLGA) and modified with rhTSH (^131^I-PLGA-rhTSH), was a feasible avenue for the integration of ^131^I and rhTSH. Meanwhile, the encapsulation efficiency (EF) of ^131^I-PLGA-rhTSH nanoparticles was approximately 60%, and the radioactivity of a single nanoparticle was about 1.1×10-2 Bq. Meanwhile, the ^131^I-PLGA-rhTSH nanoparticles were selectively delivered into, gradually enriched and slowly downregulated in xenograft tumor after the administration of ^131^I-PLGA-rhTSH nanoparticles through tail vein in mouse tumor xenograft model. Thereafter, the tumor weight was significantly reduced after the administration of ^131^I-PLGA-rhTSH nanoparticles. Subsequently, the application of ^131^I-PLGA-rhTSH nanoparticles facilitated apoptosis and attenuated immobilization via inhibiting F-actin assembling of FTC-133 cells.

**Conclusion:**

The present study develops a suitable approach integrating ^131^I and rhTSH, and this strategy is a feasible regimen enhancing the effect of radioiodine ablation for the treatment of thyroid cancer.

## 1 Introduction

Thyroid cancer is a common malignancy of the endocrine organs with a gradual increase in morbidity (1), and has become the fifth leading cause of cancer worldwide (2). Generally, the vast majority (>90%) of thyroid cancers are differentiated thyroid cancers (DTCs), including papillary thyroid cancer (PTC) and follicular thyroid carcinoma (FTC) (2). DTC usually tends to be biologically indolent, highly curable, and has a favorable prognosis after thyroidectomy followed by internal radiotherapy using iodine-131 (^131^I) to abolish thyroid cancer metastases and remnants (1, 3, 4). However, thyroid cancer such as FTC has a chance of developing into aggressive thyroid carcinoma (ATC) with metastatic propensity and resistance to radioiodine; that is, thyroid cancer will become indolent and endow a worse outcome when the thyroid cancer cells lose radioiodine avidity (1, 2, 5). It is well known that the efficacy of internal radiotherapy relies on the ability of thyroid cancer cells to absorb and accumulate active iodide (1). Hence, exploring methods for potentiating the recruitment of active iodide in thyroid cancer cells is a feasible therapeutic strategy for treating ATC with metastases and its remnants.

The sodium/iodide symporter (NIS) encoded by SLC5A5 is a transmembrane glycoprotein located on the basement membrane of thyroid follicular cells and is responsible for delivering iodine into thyroid follicular cells to synthesize hormones (2, 6). NIS transports ^131^I into thyroid carcinoma to trigger the apoptosis of thyroid cancer cells when administering internal radiotherapy using ^131^I (7). However, the expression of NIS is downregulated in ATC, and the reduced expression of NIS usually limits radioiodine uptake, which contributes to the poor prognosis of ATC (7–9). Herein, exploring approaches to increase the NIS expression is a feasible strategy to increase radioiodine intake in the thyroid cancer cells, thereafter inhibiting metastatic propensity and erasing remnants of ATC. Intriguingly, the administration of recombinant human thyroid-stimulating hormone (rhTSH) could exert multiple effects to improve the quality of life for patients with ATC by promoting *de novo* NIS synthesis (10). Meanwhile, an increasing number of studies indicate that the expression of the thyroid-stimulating hormone receptor (TSHR), a glycoprotein receptor, is more persistent than other differentiation markers in the resected thyroid tissues after thyroidectomy, including thyroglobulin (TG) proteins and NIS (3, 11, 12), suggesting that the reason for the beneficial effect of rhTSH application for treating ATC can be attributed to coupling TSHR. With the coupling of rhTSH and TSHR, the downstream pathways, such as the cyclic adenosine monophosphate (cAMP)/protein kinase A (PKA) pathway and protein kinase B (PKB)/mitogen-activated protein kinase (MAPK) pathway (13), are activated or inactivated to accumulate radioiodine to abolish cancer cells in ATC (14, 15). Hence, the integration of ^131^I with rhTSH might produce a targeted drug delivery system and initiate multifaceted effects for treating ATC. A new question is issued, namely, how to integrate ^131^I and rhTSH.

Nanomedicine as drug carriers using nanoparticles encapsulated with ^131^I and fabricated with rhTSH might provide an answer to this question. Poly (lactic-co-glycolic acid) (PLGA) is biocompatible and biodegradable and has been widely used for nanoparticles delivering miRNAs and drugs (16–19). A previous study demonstrated that boron neutron capture therapy (BNCT) using PLGA is a powerful and selective anti-cancer therapy using ^10^B-enriched boron drugs (20), implying that ^131^I is a suitable candidate for establishing PLGA-encapsulated ^131^I nanoparticles (^131^I-PLGA). Meanwhile, the nanoparticles conjugated with anti-HER2 affibodies induce the selective binding of nanoparticles to HER2-overexpressing breast cancer cells through an antibody-to-antigen pattern (21), suggesting that the nanoparticles of ^131^I-PLGA modified by rhTSH, as an antigen, were selectively bound to TSHR-expressing thyroid cancer cells in an antibody-to-antigen manner. Thence, we hypothesized that PLGA-encapsulated ^131^I nanoparticles modified by rhTSH (^131^I-PLGA-rhTSH) used in this study facilitated ^131^I delivery into thyroid cancer cells to initiate radioiodine ablation by increasing apoptosis. This study certifies the feasibility of ^131^I-PLGA-rhTSH and its effect on intensifying radioiodine ablation for treating thyroid cancer.

## 2 Materials and methods

### 2.1 Synthesis of ^131^I-PLGA nanoparticles

A double-emulsion solvent evaporation technique was performed to establish the nanoparticles. In brief, poly (lactide-co-glycolide) with acid-terminated groups (250 mg, Resomer^®^ 502H, D,l-lactide/glycolide = 50:50 mol/mol; Mw 7,000–17, 000 Da, η 0.16–0.24 dl/g, Sigma-Aldrich, Munich, Germany) was dissolved in 5 ml of dichloromethane (DCM). ^131^I sodium solution (3,700 MBq) was purchased from Gaotong Isotope (Chengdu, China). The solutions were thoroughly mixed and emulsified with a high-shear mixer (FJ300-SH, Huxi, Shanghai, China), to obtain the primary emulsion (w/o) with a ^131^I-to-PLGA polymer ratio of 1:10 (w/w). After that, the primary emulsion was added to a 1% solution of polyvinyl alcohol (PVA, 9,000–10,000 Da, Sigma-Aldrich, Munich, Germany) in phosphate buffer saline (PBS, pH ~7.4) and emulsified with a high-shear mixer (FJ300-SH, Huxi, Shanghai, China) followed by high-pressure homogenization at 1,000 bar (10 min, PandaPLUS 2000, GEA, Parma, Italy). Then, the samples were passed through a microfluidizer (M700, Microfluidics, Newton, MA, USA) for 3 min. The phase volume ratio of the double emulsion was 2:3:25, and the organic solvent was removed under vacuum (25 mbar) using a rotary evaporator. Subsequently, the suspension was filtered through a glass porous filter and freeze-dried with 5% mannitol as a cryoprotectant. Finally, the lyophilized ^131^I-PLGA nanoparticles were stored at 4°C.

### 2.2 Establishment of ^131^I-PLGA-rhTSH nanoparticles

The ^131^I-PLGA nanoparticles were dispersed and dissolved in 2-(N-Morpholino) ethanesulfonic acid (MES) buffer (pH 6.0). Then, the suspension was mixed with EDC and NHS and oscillated on a shaker for 30 min at room temperature. After that, the specimen was centrifuged at 3,000 rpm after being washed with ddH_2_O three times. Subsequently, the sample was dissolved in 0.1-M MES buffer (pH 8.0) containing 5 μg/ml rhTSH (GP21319, GLPbio, Montclair, CA, USA) and vibrated on a shaker for 2 h at room temperature to obtain ^131^I-PLGA-rhTSH nanoparticles after being rinsed with ddH_2_O three times.

### 2.3 Scanning electron microscopy

The procedures for SEM were performed as previously described (22). Briefly, ^131^I-PLGA-rhTSH nanoparticles were loaded onto coverslips. Afterward, the samples were dehydrated with gradient alcohol and stained with tertiary butyl alcohol from 50% to 100%. After that, the specimens were coated with gold under vacuum for 1 min in the chamber of a sputter coater. Then, the surface of the nanoparticles was visualized by a scanning electron microscope (Crossbeam 340, Carl Zeiss, Weimar, Germany) at an accelerating voltage of 30 kV.

### 2.4 Autoradiography

The PLGA and ^131^I-PLGA-rhTSH nanoparticles were mounted on glass slides and wrapped in 10% neutral balsam. Then, the glass slides were placed vertically to drain the excessive nuclear emulsion (Hypercoat™ EM-1, GE, Boston, USA) off after they were immersed in nuclear emulsion. Then, the specimens were exposed for 3 days after being put into the X-ray cassette and dried with silica gel at 4°C. Subsequently, the D-19 developer (RP X-OMAT LO, Kodak, Rochester, USA) was applied to develop the ^131^I in ^131^I-PLGA nanoparticles for 2 min at 20°C. Afterward, the samples were fixed with F-5 fixer (RP X-OMAT, Kodak, Rochester, USA) and rinsed with ddH_2_O three times. Finally, the deposition of black silver particles in ^131^I-PLGA-rhTSH nanoparticles on the glass slides was observed by a light microscope to understand the encapsulation of radionuclides in the nanoparticles.

### 2.5 Immunofluorescence staining

The ^131^I-PLGA-rhTSH nanoparticles were mounted on glass slides and fixed with 4% paraformaldehyde (PFA) in 0.01 M PBS for 30 min at room temperature. Then, the samples were incubated with anti-TSHR primary antibodies (Abcam, Cambridge, UK) or anti-cleaved caspase-3 primary antibodies (Cell Signaling Technology, Danvers, MA, USA) overnight at 4°C. On the following day, the samples were immersed in Alexa Fluor^®^ 555-conjugated secondary antibody (1:100, Beyotime, Shanghai, China) for 2 h at room temperature after being washed with PBS. Subsequently, 4′-6-diami-dino-2-phenylindole (DAPI; Beyotime, Shanghai, China) was used to counterstain cell nuclei for 10 min at room temperature. The images were captured with a fluorescence microscope (Carl Zeiss, Weimar, Germany) and analyzed with ImageJ software (ImageJ 1.8, NIH, USA).

### 2.6 Measurement of the nanoparticle diameter

The lyophilized nanoparticles were resuspended in purified, deionized (Milli-Q) water. Then, the polydispersity index (PDI) and average diameter of the nanoparticles were determined using a Mastersizer 3000 (Malvern, Cambridge, UK). All measurements were carried out in triplicate with a 50-fold dilution of the suspension.

### 2.7 Evaluation of ^131^I content and encapsulation efficiency of ^131^I-PLGA-rhTSH nanoparticles

The lyophilized nanoparticles were resuspended in purified deionized (Milli-Q) water and rinsed 20 times. Then, all the deionized water was collected, and the radioactivity was determined by a radioactivity meter (CRC-15R, Capintec, NJ, USA) after being centrifuged at 3,000 rpm. The ^131^I encapsulation efficiency (EE, %) was determined by the formula:


$$\text{EE (\%) ;in ;nanoparticles=}\frac{the total ;radioactivity of added 131I-the total radioactivity of centrifuged supernatant}{the total radioactivity of added 31I}$$

To evaluate the average radioactivity of each particle, the centrifuged nanoparticles were resuspended in purified, deionized (Milli-Q) water. Then, the number of nanoparticles was measured using a blood cell counting plate under a high-power field. After that, the radioactivity of a single nanoparticle was calculated using the formula:


$$\text{the radioactivity of a single nanoparticle=}\frac{the total radioactivity of added 131I-the total radioactivity of centrifuged ;supernatant}{\text{the total number of nanoparticles}}$$

All measurements were calculated in triplicates.

### 2.8 Measurement of radiochemical purity of ^131^I-PLGA-rhTSH nanoparticles

The ^131^I-PLGA-rhTSH nanoparticles were resuspended in 0.01 M PBS or serum and stood at 37°C for 30 h. The nanoparticles were washed with purified, deionized (Milli-Q) water 20 times. Then, all the deionized water was collected, and the radioactivity was determined by a radioactivity meter (CRC-15R, Capintec, NJ, USA) after being centrifuged at 3,000 rpm at 0, 10, 20, and 30 h. The radiochemical purity (%) was determined by the following formula:


$$\text{Radiochemical purity(\%)=}\frac{the total radioactivity of loaded 131I-the total radioactivity of centrifuged supernatant}{the total radioactivity of loaded 131I}$$

### 2.9 Cell culture

FTC-133 is a human FTC cell line (ECACC94060901) derived from lymph node metastasis. FTC-133 cells were cultured in Dulbecco’s modified Eagle’s medium/F-12 (1:1) (DMEM/F12; Hyclone, Logan, Utah, USA) supplemented with 10% (v/v) fetal bovine serum (FBS; Hyclone, Logan, Utah, USA) and 100 μg/ml streptomycin (Beyotime, Shanghai, China) and 100 IU/ml penicillin (Beyotime, Shanghai, China) in 75 cm^2^ cell culture flasks under humidified 5% CO_2_ at 37°C. After trypsinization, cells were seeded in 6-well plates for flow cytometry and in 96-well plates for cell viability assays, IF staining, and lactate dehydrogenase (LDH) release assays.

### 2.10 Cell viability determination

A total of 100 μl of FTC-133 cell suspension was seeded into 96-well plates at a density of 1 × 10^4^ cells/ml for 24 h. Afterward, the culture medium was exchanged for culture medium without any treatment (Control group), with PLGA (PLGA group), ^131^I (^131^I group), or ^131^I-PLGA-rhTSH nanoparticles (^131^I-PLGA-rhTSH group) for 1, 2, and 3 days, respectively. After that, the culture medium was replaced with fresh medium containing 10% (v/v) cell counting kit-8 (CCK-8) solution for another 3 h at 37°C. Subsequently, the absorbance value of the culture medium was measured at a test wavelength of 450 nm by a microplate reader (Varioskan, Thermo Fisher Scientific, Waltham, MA, USA), and 630 nm was used as a reference wavelength. Each experiment was repeated six times.

### 2.11 Lactate dehydrogenase release

LDH was used to assess the membrane integrity of FTC-133 cells after they were treated in different groups, as previously described (23). Briefly, a total of 100 μl of FTC-133 cell suspension was seeded into 96-well plates at a density of 1 × 10^4^ cells/ml for 24 h. Subsequently, the culture medium was replaced with culture medium without any treatment (Control group), with PLGA (PLGA group), ^131^I (^131^I group) or ^131^I-PLGA-rhTSH nanoparticles (^131^I-PLGA-rhTSH group). Then, the culture supernatants were collected, and cells in each group were lysed with 2% Triton X-100 (Sigma-Aldrich, Munich, Germany) for 15 min to release the total LDH from the cytoplasm on days 1, 2, and 3. Afterward, the total LDH released from cells was loaded as positive controls. The absorbance value was determined at a test wavelength of 450 nm with a microplate reader (Varioskan, Thermo Fisher Scientific, Waltham, MA, USA) according to the manufacturer’s instructions. The results were expressed as the percentage of LDH released in the medium compared to the total LDH in the cytoplasm. Each experiment was repeated four times.

### 2.12 Apoptosis assay

The FITC Annexin V Apoptosis Detection Kit with propidium iodide (PI) (BioLegend, San Diego, CA, USA) was used to evaluate the apoptosis of FTC-133 cells in each group according to the manufacturer’s instructions. In brief, trypsinized FTC-133 cells were dissociated into single cells after trypsinization and collected after centrifugation at a speed of 1,000 rpm on days 1, 2, and 3. After that, cells were washed with PBS and incubated in FITC Annexin V ready-to-use solution for 10 min, and PI ready-to-use solution for 5 min on ice. Afterward, the specimens were rinsed with PBS, collected, and resuspended. Finally, the apoptotic cells were determined by an ACEA NovoFlow (Omni, San Diego, CA, USA) and calculated by NovoExpress 1.2.1 (Omni, San Diego, CA, USA). Each experiment was repeated four times.

### 2.13 Boyden invasion assay

Boyden chamber invasion experiments were performed with Transwell inserts (Corning, NY, USA) with 8 µm pore size and 6.5 mm diameter, and the inserts were coated with Matrigel matrix (Corning, NY, USA). Then, a total of 1 × 10^5^ cells were seeded into the upper well in DMEM/F12 medium supplemented with 10% FBS in different groups. Meanwhile, the lower well received DMEM/F12 medium supplemented with 20% FBS as a chemoattractant. After 24 h, cells on the surface of the upper well were erased using a cotton swab. Cells in the lower well were fixed with 4% PFA in 0.01 M PBS for 20 min and incubated in a 0.1% crystal violet solution in 20% methanol for 10 min. The inserts were rinsed twice with PBS and once with Milli-Q water. Subsequently, the inserts were mounted on glass slides and sealed with 10% neutral balsam. Pictures were captured with a light microscope. Eight microscopic fields of each insert were collected, and the average number of invaded cells was analyzed using ImageJ software (ImageJ 1.8, NIH, USA).

### 2.14 Thyroid tumor xenograft model

Male BALB/c nude mice (6–8 weeks, 16–18 g) were purchased from the Animal Laboratory Center of Chongqing Medical University Animal Laboratory (Chongqing, China). This study was approved by the Chongqing Medical University Ethics Committee. All procedures were performed according to China’s animal welfare legislation for protecting animals used for scientific purposes and the ARRIVE guidelines. The mice were housed in pathogen-free conditions with a 12-hour light-dark cycle, and all mice were provided with an *ad libitum* supply of water and food. One week after adaptive feeding, the tumor model was established by a subcutaneous injection of 200 μl of sterile PBS containing 1  ×  10^6^ cells of FTC-133 *via* the right thigh, as previously described (8). The experiments were initiated when the tumor volume was more than 150 mm^3^ using the following formula: tumor volume = length × width^2^ × 0.52.

### 2.15 Biodistribution

The tissues of the tumor, blood, brain, heart, liver, spleen, lung, kidney, stomach, intestine, femur, and muscle were harvested and weighed. Radioactivity was measured by a radioactivity meter (CRC-15R, Capintec, NJ, USA), and the corresponding percentage of the injected dose per gram of tissue (%ID/g) was calculated.

### 2.16 Tumor weight determination

The experiments were initiated when the tumor volume was greater than 150 mm^3^ after the establishment of the xenograft tumor model. The mice were randomly assigned to the following groups:

1) Control group. Nude mice were injected with 100 μl of 0.9% NaCl through a tail vein.

2) PLGA group. Nude mice were injected with the same amount of PLGA in 100 μl of 0.9% NaCl through the tail vein.

3) ^131^I group. Nude mice were injected with 18.5 MBq of ^131^I sodium solution in 100 μl of 0.9% NaCl through the tail vein.

4) ^131^I-PLGA-rhTSH group. Nude mice were injected with 18.5 MBq of ^131^I-PLGA-rhTSH nanoparticles in 100 μl of 0.9% NaCl through the tail vein.

On day 21 after treatment, all the mice were euthanized. The tumor tissues were dissected, and the weight was measured by an electronic balance (Sartorius, Göttingen, Germany) by an investigator blinded to the group assignment.

### 2.17 TUNEL staining

The apoptosis of thyroid cancer cells was determined on day 21 after the establishment of the xenograft tumor model by TUNEL staining with an Apoptosis Detection Kit (Beyotime, Shanghai, China) according to the manufacturer’s instructions, as previously described (24). The number of TUNEL-positive thyroid cancer cells in the xenograft tumor was counted, and the final data were expressed as the number of TUNEL-positive thyroid cancer cells per mm^2^. The analysis was performed by an investigator blinded to group assignment.

### 2.18 F-actin assembling detection

The status of F-actin assembly in each group was determined as described previously (25). In brief, the samples from each group were incubated in 4% paraformaldehyde (PFA) for 10 min at room temperature and washed with PBS three times. Subsequently, the samples were immersed in Alexa Fluor 488 conjugated phalloidin reagents (Life Technologies, Waltham, MA, USA) at room temperature for 30 min. After being mounted on glass slides, the images were captured with a fluorescence microscope (Carl Zeiss, Weimar, Germany) and analyzed by ImageJ software (ImageJ 1.8, NIH, USA).

### 2.19 Statistical analysis

All data were expressed as the mean ± standard deviation (SD), and analyzed using SPSS 19.0 software (IBM Corp., Armonk, NY, USA). The statistical significance of the results was analyzed by one-way or two-way analysis of variance (ANOVA), followed by Tukey’s *post-hoc* test for multiple comparisons. A p value of <0.05 was considered to demonstrate a statistical difference.

## 3 Results

### 3.1 Establishment and characteristics of ^131^I-PLGA-rhTSH nanoparticles

**Figure 1:**
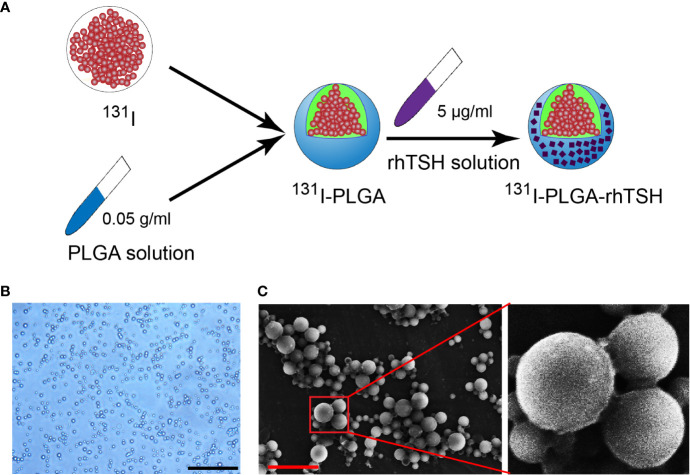
Generation of ^131^I-PLGA-rhTSH nanoparticles. **(A)** The schematic illustration of developing ^131^I-PLGA-rhTSH nanoparticles-encapsulated with ^131^I using poly (lactic-co-glycolic acid) (PLGA) and decorated with rhTSH. **(B)** The phase-contrasted images illustrating the morphology of the ^131^I-PLGA-rhTSH nanoparticles. Scale bar: 5 μm. **(C)** Scanning electron microscopy (SEM) images presenting the morphology of ^131^I-PLGA-rhTSH nanoparticles. Scale bar: 1 μm.

**Figure 2:**
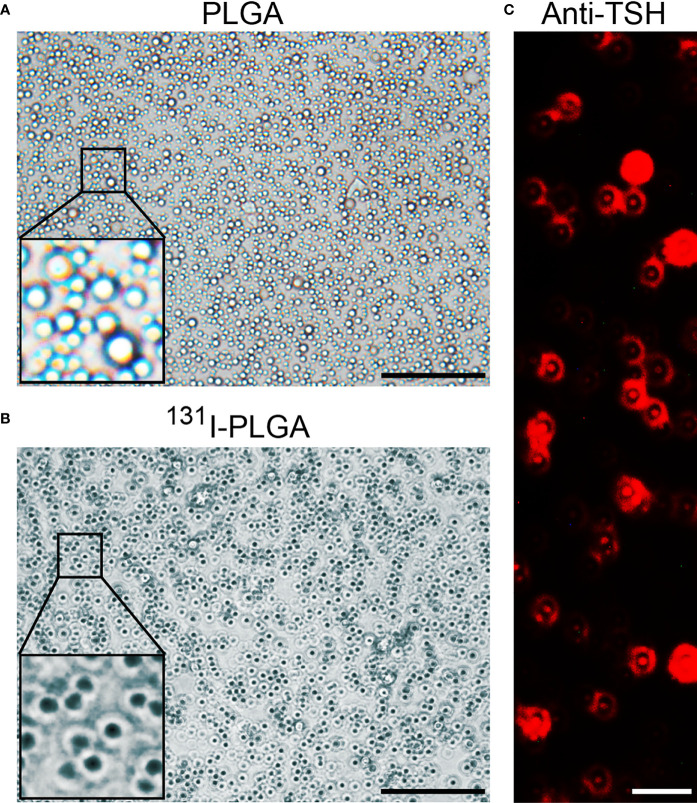
The characteristics of ^131^I-PLGA-rhTSH nanoparticles. **(A)** The autoradiography image representing the deposition of black silver particles in PLGA nanoparticles. Scale bar: 5 μm. **(B)** The autoradiography image demonstrating the accumulation of black silver particles in ^131^I-PLGA-rhTSH nanoparticles. Scale bar: 5 μm. **(C)** The immunostaining of TSH on the surface of ^131^I-PLGA-rhTSH nanoparticles.

As shown in **Figure 1A**, the ^131^I-PLGA-rhTSH nanoparticles were first synthesized using ^131^I and PLGA, then modified with rhTSH. The morphology of the ^131^I-PLGA-rhTSH nanoparticles was round-like under light microscopy (**Figure 1B**). Subsequently, SEM images indicated that most of the ^131^I-PLGA-rhTSH nanoparticles were round and the diameter was approximately 250 nm (**Figure 1C**). To validate whether ^131^I was integrated into the ^131^I-PLGA-rhTSH nanoparticles, autoradiography was performed. In the representative images presented, there was no deposition of black silver particles in the center of the PLGA nanoparticles, whereas the accumulation of black silver particles was observed in the center of the ^131^I-PLGA-rhTSH nanoparticles (**Figures 2A, B**). After that, immunostaining of TSH was conducted to verify the absorbance of TSH on the surface of the ^131^I-PLGA-rhTSH nanoparticles. The immunostaining images exhibited that nearly each nanoparticle was surrounded by a round circle on the surface of the ^131^I-PLGA-rhTSH nanoparticles (**Figure 2C**). Collectively, these results demonstrated that the constructed nanoparticles finely encapsulated ^131^I in PLGA nanoparticles, and rhTSH was absorbed on the surface of the ^131^I-PLGA-rhTSH nanoparticles, which served as a selective guide for TSHR-expressing thyroid cancer.

**Figure 3:**
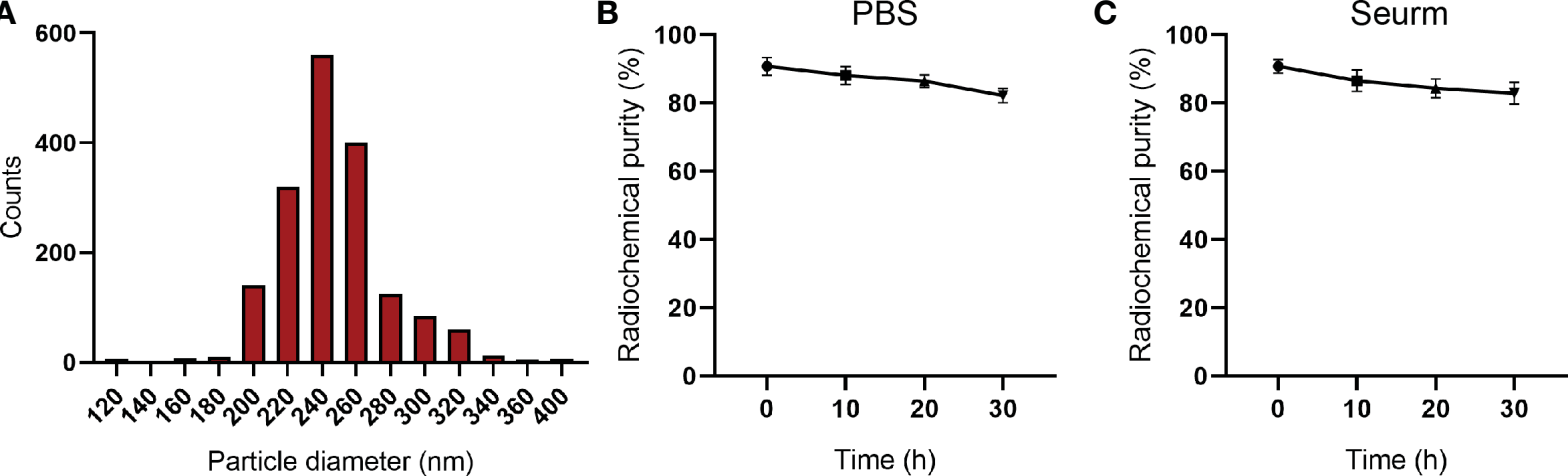
The diameter and radiochemical purity of ^131^I-PLGA-rhTSH nanoparticles. **(A)** The diameter of the ^131^I-PLGA-rhTSH nanoparticles. **(B)** The radiochemical purity in PBS at 10 h, 20 h, and 30 h. **(C)** The radiochemical purity of serum at 10 h, 20 h, and 30 h.

Next, the diameter of the ^131^I-PLGA-rhTSH nanoparticles was assessed, and the results showed that the diameter of the ^131^I-PLGA-rhTSH nanoparticles ranged from 200 nm to 320 nm, with an average diameter of 243.7 ± 0.25 nm (**Figure 3A**). After that, radiochemical purity was assessed in PBS and serum at different timepoints (10 h, 20 h, and 30 h). Furthermore, the curve illustrated that the radiochemical purity slowly declined in PBS and serum with time, and the average radiochemical purity was above 80% (**Figures 3B, C**). These results illustrate that the ^131^I-PLGA-rhTSH nanoparticles were biologically stable *in vitro*.

### 3.2 The encapsulation efficiency of ^131^I in ^131^I-PLGA-rhTSH nanoparticles

The EF was performed to evaluate the efficacy of ^131^I encapsulated in ^131^I-PLGA-rhTSH nanoparticles. The results indicated that the EF was 61.17 ± 1.31 and the radioactivity of a single nanoparticle was 1.1 ± 0.12 × 10^−2^ Bq (**Table 1**). After that, the approximate number of ^131^I-PLGA-rhTSH nanoparticles used *in vitro* and *in vivo* experiments was calculated using the formula: nanoparticle number = the total radioactivity/the radioactivity of a single nanoparticle (1.1 × 10^−2^ Bq).

**Table 1:** Encapsulation efficiency (EF) of ^131^I and the radioactivity of a single nanoparticle.

Encapsulation efficiency (EF) of ^131^I (%)	Radioactivity (Bq)
61.17 ± 1.31	1.1 ± 0.12 × 10^−2^

### 3.3 The administration of ^131^I-PLGA-rhTSH nanoparticles significantly reduced tumor weight by triggering apoptosis in thyroid cancer

The above findings demonstrated that the ^131^I-PLGA-rhTSH nanoparticles were biologically stable *in vitro* and whether the ^131^I-PLGA-rhTSH nanoparticles held the potential for targeted delivery into tumor tissue. The mouse tumor xenograft model was established and the ^131^I-PLGA-rhTSH nanoparticles were injected through the rail vein. The biodistribution of ^131^I-PLGA-rhTSH in nude mice with thyroid cancer was investigated. The results indicated that the percentage of injected dose per gram of tissue (%ID/g) of ^131^I-PLGA-rhTSH was higher in blood, liver, spleen, lung, and kidney from 12 h to 48 h, whereas that was obviously lower at 72 h (**Table 2**). However, the %ID/g in tumor tissue showed a slowly downregulated tendency (**Table 2**). Furthermore, the %ID/g was very low in the brain, stomach, intestine, femur, and muscle (**Table 2**).

**Table 2:** Biodistribution of ^131^I-PLGA-rhTSH in nude mice with thyroid cancer (% ID/g).

Location	12 h	24 h	48 h	72 h
Tumor	5.36 ± 0.41	4.97 ± 0.53	4.11 ± 0.31	3.71 ± 0.28
Blood	14.77 ± 1.88	7.29 ± 0.71	6.01 ± 0.66	2.27 ± 0.27
Brain	0.68 ± 0.11	0.49 ± 0.17	0.38 ± 0.13	0.14 ± 0.08
Heart	3.79 ± 0.67	1.77 ± 0.45	1.06 ± 0.22	0.87 ± 0.12
Liver	9.78 ± 0.43	6.93 ± 0.86	4.74 ± 0.88	3.29 ± 0.45
Spleen	6.03 ± 1.16	3.69 ± 0.45	1.79 ± 0.23	0.78 ± 0.14
Lung	5.92 ± 0.69	4.66 ± 0.29	3.24 ± 0.76	1.04 ± 0.23
Kidney	6.23 ± 0.82	6.03 ± 0.71	5.04 ± 0.55	3.31 ± 0.39
Stomach	2.69 ± 0.84	1.76 ± 0.69	1.43 ± 0.29	1.06 ± 0.21
Intestine	2.02 ± 0.82	1.84 ± 0.65	1.03 ± 0.21	0.97 ± 0.18
Femur	0.81 ± 0.18	0.69 ± 0.07	0.44 ± 0.08	0.13 ± 0.04
Muscle	1.47 ± 0.38	0.90 ± 0.21	0.83 ± 0.13	0.12 ± 0.06

**Figure 4:**
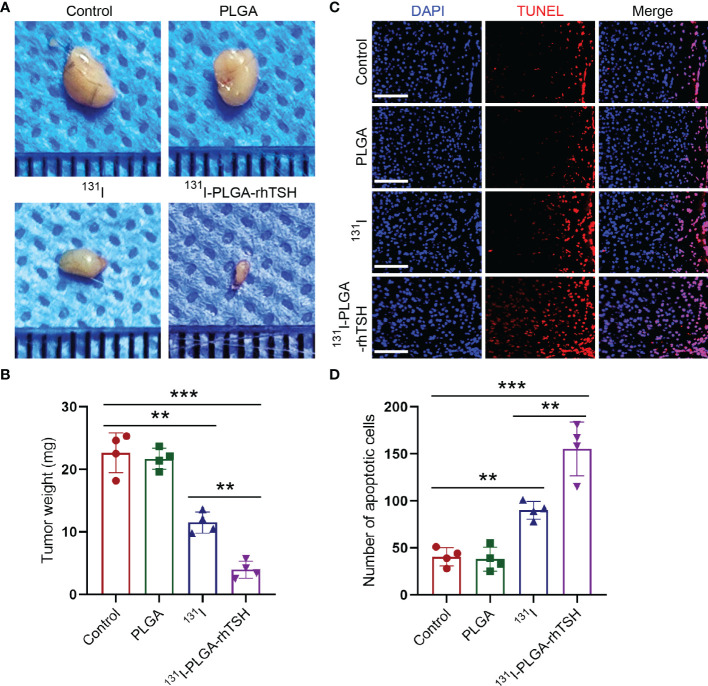
The application of ^131^I-PLGA-rhTSH nanoparticles reduced tumor size through inducing apoptosis in thyroid cancer. **(A)** The representative images illustrating the tumor size in each group. **(B)** A summarized bar graph presenting the tumor weight from **(A)**. n = 4 each group, ^**^ *p <*0.01, ^***^ *p <*0.001; one-way ANOVA, followed by Tukey’s *post hoc* test. **(C)** The typical images revealing the number of apoptotic cells in each group. Scale bars: 20 μm. **(D)** Summarizing bar graph illustrating the number of apoptotic cells from **(C)**. n = 4 each group, ^**^ *p <*0.01, ^***^ *p <*0.001; one-way ANOVA, followed by Tukey’s *post hoc* test.

Considering that the therapeutic strategy of radioiodine ablation using ^131^I would take effect on day 21 (26, 27), the weight of thyroid cancer was assessed on day 21 in different groups. The results illustrated that the tumor weight was reduced with the administration of ^131^I (**Figures 4B, C**), whereas that was significantly reduced with the application of ^131^I-PLGA-rhTSH nanoparticles (**Figures 4A, B**). Meanwhile, TUNEL staining showed that the number of apoptotic cells was significantly increased with the administration of ^131^I-PLGA-rhTSH nanoparticles (**Figures 4C, D**). Furthermore, the number of apoptotic cells in ^131^I-PLGA-rhTSH group was larger than that in ^131^I group, PLGA group and Control group (**Figures 4C, D**). These results revealed that the administration of ^131^I-PLGA-rhTSH nanoparticles significantly diminished the tumor weight by inducing apoptosis in thyroid cancer cells, indicating that the application of ^131^I-PLGA-rhTSH nanoparticles is a feasible candidate for treating thyroid cancer.

### 3.4 The administration of ^131^I-PLGA-rhTSH nanoparticles increased LDH release and reduced cell viability

**Figure 5:**
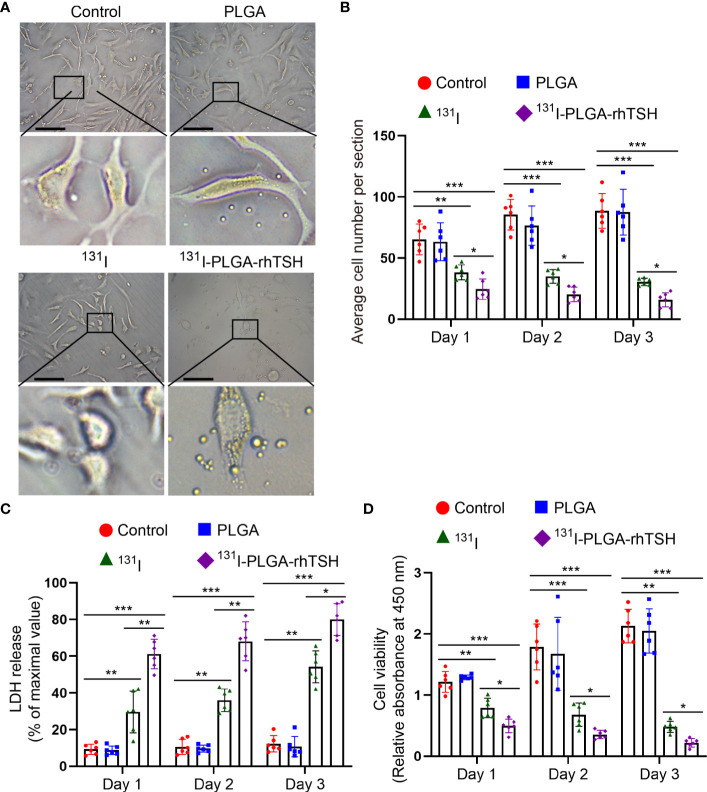
The administration of ^131^I-PLGA-rhTSH nanoparticles increased LDH release and downregulated cell viability. **(A)** The typical images showcasing the condition of FTC-133 cells in each group. Scale bars: 20 μm. **(B)** A quantitative analysis of the average cell number per section in each group from **(B)**. n = 6 per group, ^*^ *p <*0.05, ^**^ *p <*0.01, ^***^ *p <*0.001; two-way ANOVA, followed by Tukey’s *post hoc* test. **(C)** A bar graph demonstrating the LDH release in each group. n = 6 per group, ^*^ *p <*0.05, ^**^ *p <*0.01, ^***^ *p <*0.001; two-way ANOVA, followed by Tukey’s *post hoc* test. **(D)** A bar chart summarizing the relative absorbance value at 450 nm using the CCK-8 assay in each group. n = 6 per group, ^*^ *p <*0.05, ^**^ *p <*0.01, ^***^ *p <*0.001; two-way ANOVA, followed by Tukey’s *post hoc* test.

To further uncover the reason why the administration of the ^131^I-PLGA-rhTSH nanoparticles decreased tumor weight; FTC-133 cells were exposed to different treatments. The phase-contrast images showed that few PLGA nanoparticles were in the cytoplasm, whereas the ^131^I-PLGA-rhTSH nanoparticles emerged in the cytoplasm and appeared to have a clustered distribution (**Figure 5A**). Furthermore, the results showed that the average cell number in the ^131^I and ^131^I-PLGA-rhTSH groups evidently decreased on days 1, 2, and 3, compared with the Control and PLGA groups (**Figure 5B**). Furthermore, the administration of ^131^I-PLGA-rhTSH nanoparticles showed a better effect than that in the ^131^I group (**Figure 5B**). Subsequently, the LDH-release level demonstrated that the percentage of LDH release to the maximal value was prominently upregulated in groups ^131^I and ^131^I-PLGA-rhTSH on days 1, 2, and 3, compared with groups Control and PLGA (**Figure 5C**). At the same time, the percentage was highest in the ^131^I-PLGA-rhTSH group among the four groups (**Figure 5C**). Additionally, the cell viability in each group was assessed using CCK-8 assays, and the results exhibited the same tendency as the average cell number (**Figure 5D**). Meanwhile, the application of ^131^I-PLGA-rhTSH exerted the best anti-tumor effects (**Figure 5D**). Taken together, these results illustrate that ^131^I-PLGA-rhTSH nanoparticles could enrich the cytoplasm to evoke an anti-tumor effect.

### 3.5 The application of ^131^I-PLGA-rhTSH nanoparticles facilitated thyroid cancer cells apoptosis and immobilization *in vitro*

**Figure 6:**
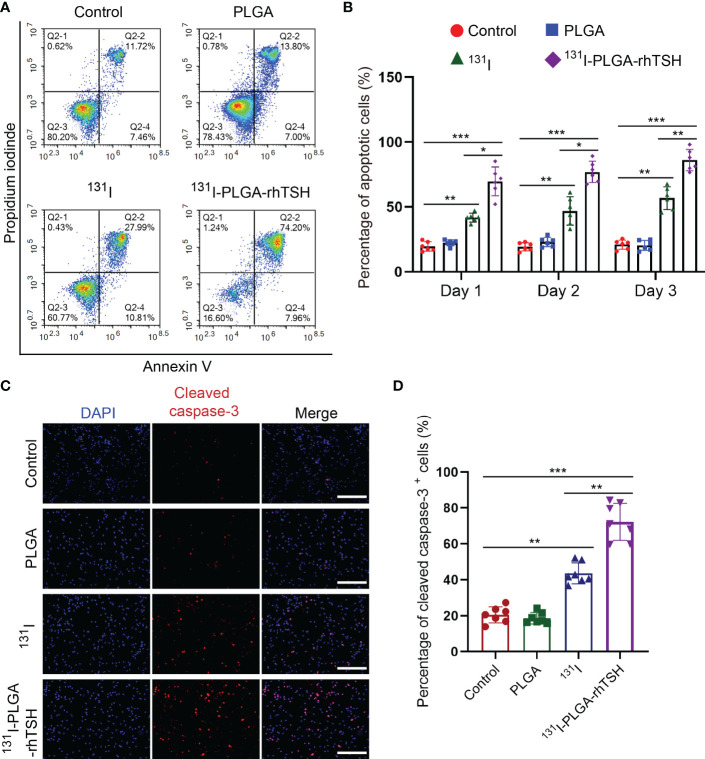
The application of ^131^I-PLGA-rhTSH promoted the apoptosis of FTC-133 cells. **(A)** The flow cytometry images revealing the cell distribution in different groups on day 3. **(B)** A summarized bar graph showing the percentage of apoptotic cells from **(A)**. n = 6 per group, ^*^ *p <*0.05, ^**^ *p <*0.01, ^***^ *p <*0.001; two-way ANOVA, followed by Tukey’s *post hoc* test. **(C)** The typical immunostaining images of cleaved caspase-3 in each group on day 3. The cell nuclei were counterstained with DAPI in blue. Scale bars: 50 μm. **(D)** A bar chart summarizing the percentage of cleaved caspase-3^+^ cells in each group. n = 7 per group; ^**^ *p <*0.01, ^***^ *p <*0.001; one-way ANOVA, followed by Tukey’s *post hoc* test.

**Figure 7:**
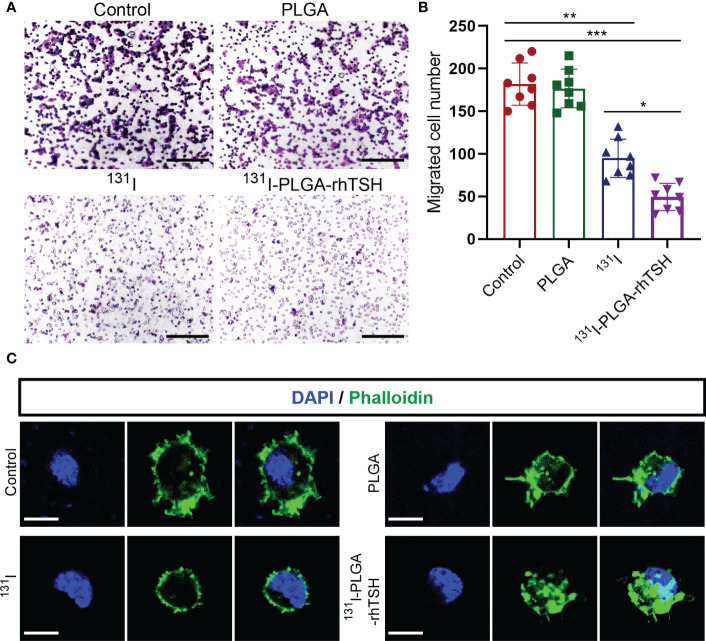
The application of ^131^I-PLGA-rhTSH inhibited FTC-133 cells mobilization through reducing F-actin assembling. **(A)** The typical transwell images illustrating the mobilization ability of FTC-133 cells per group. Scale bars: 50 μm. **(B)** Bar graph summarizing the migrated cell number from upper to lower chamber from **(A)**. n = 8 per group; ^*^ *p <*0.05, ^**^ *p <*0.01, ^***^ *p <*0.001; one-way ANOVA, followed by Tukey’s *post hoc* test. **(C)** The representative phalloidin staining images presenting the status of F-actin assembly in each group. Scale bars: 5 μm.

To uncover the reason why ^131^I-PLGA-rhTSH nanoparticles induce the death of thyroid cancer cells, apoptosis assays were implemented. The results indicated that the proportion of apoptotic cells was significantly elevated in the ^131^I and ^131^I-PLGA-rhTSH groups on days 1, 2, and 3, compared with the Control and PLGA groups (**Figures 6A, B**). Furthermore, the administration of ^131^I-PLGA-rhTSH ignited a higher percentage of apoptotic cells than that in the ^131^I group (**Figures 6A, B**). Meanwhile, the immunostaining images indicated the number of cleaved caspase-3^+^ cells was larger in groups ^131^I and ^131^I-PLGA-rhTSH than in groups Control and PLGA (**Figures 6C, D**). Furthermore, a greater number of cleaved caspase-3^+^ cells appeared in the ^131^I-PLGA-rhTSH group than that in the ^131^I group (**Figures 6C, D**). Moreover, the transwell assays depicted that the cell number penetrated from the upper to lower chambers and was profoundly reduced in the ^131^I and ^131^I-PLGA-rhTSH groups (**Figures 7A, B**). Also, the cell number was smaller in the ^131^I-PLGA-rhTSH group than in the ^131^I group (**Figures 7A, B**). Additionally, the phalloidin staining images illustrated that the F-actin assembly was markedly decreased with the administration of ^131^I and ^131^I-PLGA-rhTSH nanoparticles, and the application of ^131^I-PLGA-rhTSH nanoparticles demonstrated a better effect than that in the ^131^I group (**Figure 7C**). Collectively, these results illustrated that the application of ^131^I-PLGA-rhTSH nanoparticles facilitated thyroid cancer cell apoptosis and immobilization by inhibiting F-actin assembly *in vitro*.

## 4 Discussion

ATC usually loses radioiodine avidity resulting from NIS downregulation. The antibody-to-antigen pattern has been shown to be a suitable strategy for binding therapeutic molecules to targeted tissue for treating breast cancer (21). The expression of TSHR is more persistent than that of NIS (3), which provides a therapeutic target for ATC. Here, the integration of ^131^I and rhTSH is a promising strategy for treating ATC. Here, our results showed that the nanoparticles constructed in this study were feasible for integrating ^131^I and rhTSH. Meanwhile, the EF of ^131^I-PLGA-rhTSH nanoparticles was approximately 60%, and the radioactivity of a single nanoparticle was about 1.1 × 10^−2^ Bq. After that, the results demonstrated that the ^131^I-PLGA-rhTSH nanoparticles were successfully delivered into xenograft tumor, gradually enriching and slowly downregulating in thyroid carcinoma after the establishment of a tumor xenograft model in nude mice. At the same time, the tumor weight was significantly reduced owing to igniting apoptosis on day 21 after the administration of ^131^I-PLGA-rhTSH nanoparticles *in vivo*. Additionally, the results indicated that the application of ^131^I-PLGA-rhTSH nanoparticles elevated LDH release and decreased cell viability through upregulating FTC-133 cell apoptosis. Meanwhile, the mobilization of FTC-133 cells was obviously suppressed by reducing F-actin assembly *in vitro*. Collectively, these results showcase that the approach used to develop nanoparticles encapsulating ^131^I using PLGA and modified by rhTSH is a feasible regimen for enhancing the effect of radioiodine ablation for the treatment of thyroid cancer.

Here, the modified nanoparticles fabricated by rhTSH promote ^131^I transport into thyroid cancer cells. The reasons for this phenomenon may be multifaceted, including, but not limited to, enhancing the selective binding of rhTSH to TSHR-expressing thyroid cancer cells. Targeted therapy using antibodies binding to antigen has been widely used for anti-angiogenetic regimens with bevacizumab to treat various cancers, including non-small-cell lung cancer, metastatic breast cancer, colorectal cancer, renal cell carcinoma, glioblastoma, ovarian cancer and cervical cancer (28–31). With the binding of an antibody to an antigen, a series of downstream cascades are activated to induce apoptosis and inhibit proliferation of cancer cells (32–34), which is consistent with our results that more thyroid cancer cells suffer from apoptosis with the coupling of rhTSH to TSHR. And, the main mediator resulting in apoptosis might be due to the conjugated ^131^I because the administration of rhTSH could exert multiple effects to improve the quality of life for patients with ATC, including promoting *de novo* NIS and thyroglobulin (TG) synthesis, organificating radioiodine in the nascent TG, elongating radioiodine half-life in the remnant, lowering the amount of TG in the bloodstream and reducing radioiodine biodistribution in other organs (10).

NIS is an effective mediator that facilitates active iodide deposition into thyroid follicular cells (8), and the upregulation of NIS promotes ^131^I transport into tumor cells (2). ^131^I accumulation induces thyroid cancer apoptosis, which agrees with the results in this study, through upregulating B-cell translocation gene 2-mediated activation of JNK/NF-κB pathways (35). Next, ^131^I deposition suppresses thyroid cancer proliferation by promoting cell cycle arrest and inhibiting BCL-2 expression (35, 36). Furthermore, the application of ^131^I evokes thyroid cancer cell immobilization, which conforms to a previous study that found that the administration of ^131^I represses thyroid cancer cell migration by modulating NIS expression and location (37). Moreover, the administration of ^131^I upregulates the secretion of tumor necrosis factor-α (TNF-α) and its receptors TNFR1 and TNFR2 to initiate anti-tumor effects (38).

A limitation of this study was that the expression of NIS and TSHR was not examined. Moreover, the molecular mechanism of apoptosis-related factors should be further elucidated to discover more therapeutic targets for treating thyroid cancer. Moreover, the method of establishing ^131^I-PLGA-rhTSH nanoparticles should be modified to make this biomaterial more effective.

## 5 Conclusion

In conclusion, our results demonstrate that the method of developing nanoparticles-encapsulated ^131^I using PLGA and decorated with rhTSH (^131^I-PLGA-rhTSH) is a feasible avenue for integrating ^131^I and rhTSH. Meanwhile, the administration of ^131^I-PLGA-rhTSH nanoparticles selectively delivered into xenograft tumor, gradually enriches, and slowly downregulates thyroid carcinoma, after that reducing tumor weight *via* inducing apoptosis in xenograft tumor model. Furthermore, the application of ^131^I-PLGA-rhTSH nanoparticles promotes apoptosis and immobilization by attenuating the F-actin assembly of thyroid cancer cells. This study provides a rationale for strengthening the effect of radioiodine ablation in treating thyroid cancer.

## Data availability statement

The original contributions presented in the study are included in the article/supplementary material. Further inquiries can be directed to the corresponding author.

## Ethics statement

This study was approved by the Chongqing Medical University Ethics Committee and all procedures were performed according to China’s animal welfare legislation for protection of animals used for scientific purpose and ARRIVE guidelines.

## Author contributions

GY designed the experiments. YF performed most of the experiments. YF and YX analyzed the results and edited figures. XW and JC performed thyroid tumor xenograft model and tail vein injection. DF and JH conducted immunostaining and FCM. YF wrote preliminary draft of the manuscript. GY revised the manuscript. All authors contributed to the article and approved the submitted version.
